# Hypomethylated RRBP1 Potentiates Tumor Malignancy and Chemoresistance in Upper Tract Urothelial Carcinoma

**DOI:** 10.3390/ijms22168761

**Published:** 2021-08-16

**Authors:** Hao-Lun Luo, Hui-Ying Liu, Yin-Lun Chang, Ming-Tse Sung, Po-Yen Chen, Yu-Li Su, Chun-Chieh Huang, Jei-Ming Peng

**Affiliations:** 1 Department of Urology, Kaohsiung Chang Gung Memorial Hospital and Chang Gung University College of Medicine, Kaohsiung 83301, Taiwan; alesy1980@gmail.com (H.-L.L.); ying_1011@hotmail.com (H.-Y.L.); tailanylyl@gmail.com (Y.-L.C.); patrick7613@gmail.com (P.-Y.C.); 2 Department of Pathology, Kaohsiung Chang Gung Memorial Hospital and Chang Gung University College of Medicine, Kaohsiung 83301, Taiwan; mtsmts@cgmh.org.tw; 3 Department of Hematology and Oncology, Kaohsiung Chang Gung Memorial Hospital and Chang Gung University College of Medicine, Kaohsiung 83301, Taiwan; yolisu@mac.com; 4 Department of Radiation Oncology, Kaohsiung Chang Gung Memorial Hospital and Chang Gung University College of Medicine, Kaohsiung 83301, Taiwan; cgukinace@gmail.com; 5 Institute for Translational Research in Biomedicine, Kaohsiung Chang Gung Memorial Hospital, Kaohsiung 83301, Taiwan

**Keywords:** upper tract urothelial carcinoma, hypomethylation, RRBP1, oncogene, patient-derived organoid, chemoresistance

## Abstract

Ribosome-binding protein 1 (RRBP1) is a potential oncogene in several cancer types. However, the correlation between RRBP1 expression and the prognosis of patients with upper tract urothelial carcinoma (UTUC) remains unclear. In this study, we identified that RRBP1 is associated with carcinogenesis and metastasis in UTUC using a methylation profiling microarray. High correlations between RRBP1 and cancer stages, nodal metastasis status, molecular subtypes, and prognosis in bladder urothelial cancer (BLCA) were found. Aberrant DNA methylation in the gene body region of RRBP1 was determined in UTUC tissues by methylation-specific PCR. RRBP1 expression was significantly increased in UTUC tissues and cell lines, as determined by real-time PCR and immunohistochemistry. RRBP1 depletion significantly reduced BFTC909 cell growth induced by specific shRNA. On the other hand, molecular subtype analysis showed that the expression of RRBP1 was associated with genes related to cell proliferation, epithelial–mesenchymal transition, and basal markers. A patient-derived organoid model was established to analyze patients’ responses to different drugs. The expression of RRBP1 was related to chemoresistance. Taken together, these results provide the first evidence that RRBP1 gene body hypomethylation predicts RRBP1 high expression in UTUC. The data highlight the importance of RRBP1 in UTUC malignancy and chemotherapeutic tolerance.

## 1. Introduction

Upper tract urothelial carcinoma (UTUC) is a cancer that is difficult to detect at an early stage, when it involves the renal pelvis and ureter [1]. The incidence of UTUC accounts for approximately 5% of all urothelial cancers, and invasive UTUC accounts for 60% of all UTUC patients. The 5-year cancer-specific survival (CSS) rate after the onset of early UTUC is <50% [2]. Compared with bladder cancer, the difficulty of UTUC discovery is that it is more difficult to detect in the early stages. This reflects the relative rarity of UTUC and the consequent lack of knowledge of the mechanism of its pathogenesis. Currently, radical nephroureterectomy (RNU) is the standard treatment for local UTUC [2,3]. Regarding prognostic effectiveness, 33% of RNU cases relapse within 5 years [4], indicating a high recurrence rate and poor prognosis [2,5], with a 5-year CSS rate of 73% [2,5]. Additional systemic treatment is required. However, the disadvantage of RNU is that it damages renal function and reduces eligibility for chemotherapy [6,7].

According to the treatment guidelines of the European Association of Urology, the chemotherapy method of UTUC is currently based on UCB-based treatment [8,9]. Cisplatin-based neoadjuvant chemotherapy (NAC) is regarded as the standard treatment for patients with locally advanced UTUC [10]. An increasing number of studies have shown that NAC and ACH have a certain effect [11,12]. However, due to the different responses of patients to chemotherapy, these cannot be used arbitrarily [13,14]. The advantage of URS is that the lesions can be directly observed, evaluated, and biopsied.

Several factors that can predict poor prognosis include age, tumor grade, tumor stage, lymph node metastasis, CLS, lymphatic vessel invasion, multifocal tumor size, and tumor size [9,15,16,17,18,19]. As there are few biomarkers used to evaluate UTUC, finding more effective tumor markers for evaluating the prognosis of UTUC could help predict prognosis and promote effective treatment.

Ribosome-binding protein 1 (RRBP1) is an important endoplasmic reticular protein that plays a key role in ribosomal binding and the termination of differentiation in secretory cells and tissues [20,21,22]. RRBP1 consists of 1410 amino acids, including a transmembrane domain that is essential for the transport and secretion of new proteins [23,24]. Although RRBP1 is mainly located in the endoplasmic reticulum (ER), it can also be detected in the nucleus and cytoplasm [25]. RRBP1 may be a potential oncogene. The RRBP1 is highly expressed in a variety of cancers, including lung [26], ovarian [27], prostatic [28], esophageal [22], rectal [29], and breast cancer [30,31]. RRBP1 is also a biomarker for poor prognosis in colorectal, prostate, and breast cancers. In addition, RRBP1 is associated with tumor cell growth and resistance to the unfolded protein response (UPR) [26], which might be an important milestone in tumor management and a potential prognostic biomarker for tumors. Even so, few studies have explored the mechanism by which RRBP1 regulates tumor cell growth, and its current functions are mainly related to ER pressure control. The role of RRBP1 in urothelial carcinoma remains unclear. Further studies on its expression, regulatory mechanisms, intracellular signaling pathways, cancerous tumors, degree of malignancy, and prognostic factors in urothelial carcinoma would inform further studies that could benefit clinical treatment of various cancers.

UTUC is a cancer that is difficult to detect in the early stage, with a marked propensity to invade and metastasize. Using DNA methylation microarray chip analysis, we identified the membrane protein RRBP1 as a tumor oncogene in UTUC cells, and the potential of RRBP1 as a clinical prognostic marker from The Cancer Genome Atlas (TCGA) BLCA database was further confirmed. Especially in advanced and metastatic UTUC, the expression of RRBP1 was significantly higher than that in normal tissues or early tumors. RRBP1 was highly expressed in both malignant tumors and cell lines in clinical specimens. Analysis of the expression of gene groups affected by RRBP1 in patients’ samples correlated increased RRBP1 expression with increased proliferation and metastasis-related proteins. Lee et al. established a patient-derived organoid (PDO) model for bladder cancer that could be used to simulate tumor response to drugs [32]. Using this method, we established PDO of UTUC and performed drug cytotoxicity analysis. The results showed that progressively lower expression levels of RRBP1 were associated with progressively increasing sensitivity of PDO to cytotoxicity due to cisplatin, gemcitabine, and epirubicin. Thus, high expression of RRBP1 was implicated as a potential oncogene marker in UTUC with reduced susceptibility to drugs.

## 2. Results

### 2.1. RRBP1 Is a Hypomethylated and Highly Expressed Oncogene in UTUC

**Figure 1:**
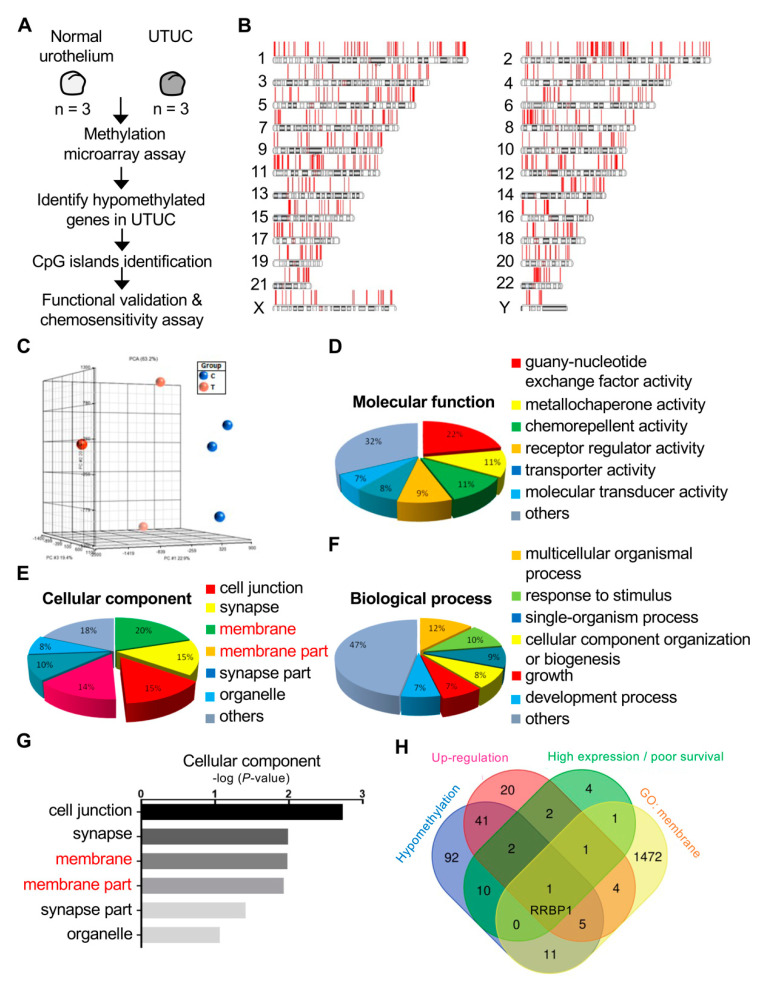
Methylation profiling analysis identified RRBP1 as an oncogene in UTUC. (**A**,**B**) The diagram shows the process of analyzing the DNA methylation profiles of normal and UTUC tumors. The DNA methylation was determined in UTUC tumor tissues from high-stage/high-grade patients and morphologically normal adjacent tissues (NAT) from low-stage/low-grade patients. The analysis of DNA methylation was conducted using a methyl-CpG binding-domain-based (MBD) protein microarray chip. (**C**–**F**) Gene oncology (GO) analysis of gene expression in molecular function, biological process, and cellular component. (**G**) Statistical analysis of gene expression in cellular component. (**H**) Venn diagram of RRBP1 satisfies the intersection conditions of hypomethylation, upregulated genes, poor survival, and cellular membrane component of GO analysis. Hypomethylation genes (133), upregulation genes (61), high expression with poor survival (5), and GO analysis: membrane (1473).

UTUC is difficult to detect at an early stage and there are few predictive factors that can be used to evaluate UTUC. Analysis of the expression level of cancer cell membrane proteins is helpful to identify applicable marker proteins and for clinical detection [33]. Previously, we identified SPARCL1 as an oncogene that promotes carcinogenesis in a bioinformatics database of UTUC provided by the National Institutes of Health (NIH) in Taiwan [34]. However, the role of membrane proteins in lymphovascular invasion in UTUC has not been clearly discussed. Thus, we further investigated the potential oncogenic factors in membrane proteins of UTUC and re-explored the NIH bioinformatics database of UTUC. Normal urothelial tissues of three patients with low-stage/low-grade UTUC were compared with three patients with high-stage/high-grade UTUC tumors from the database. A methyl-CpG binding-domain-based (MBD) protein microarray was used to compare the methylation differences between the two groups to identify the membrane proteins that showed significant differences (Figure 1A). Principal component analysis of DNA methylation revealed significant differences between normal urothelial tissue of patients with low-stage/low-grade UTUC (blue) and tumors with high-stage/high-grade UTUC (red) (Figure 1B).

The three subgroups were compared concerning molecular function, biological process, and cellular components. The membrane (15%) and membrane part (14%) of cellular components accounted for a considerable proportion and had statistical significance. The genes in the membrane were further analyzed (Figure 1C). Venn diagram analysis established the following conditions: hypomethylation of the promoters of genes in tumor tissues relative to normal tissues, high protein expression, poor survival, and membrane proteins identified from gene ontology analysis (Figure 1D). RRBP1 was the only candidate that met these criteria. Previous studies with other cancers have demonstrated that RRBP1 is highly expressed in tumors and is associated with poor prognosis. However, RRBP1 has not been reported in UTUC. On the basis of the present findings, the importance of RRBP1 in UTUC was further explored.

### 2.2. Methylation of RRBP1 in Bladder Cancer

**Figure 2:**
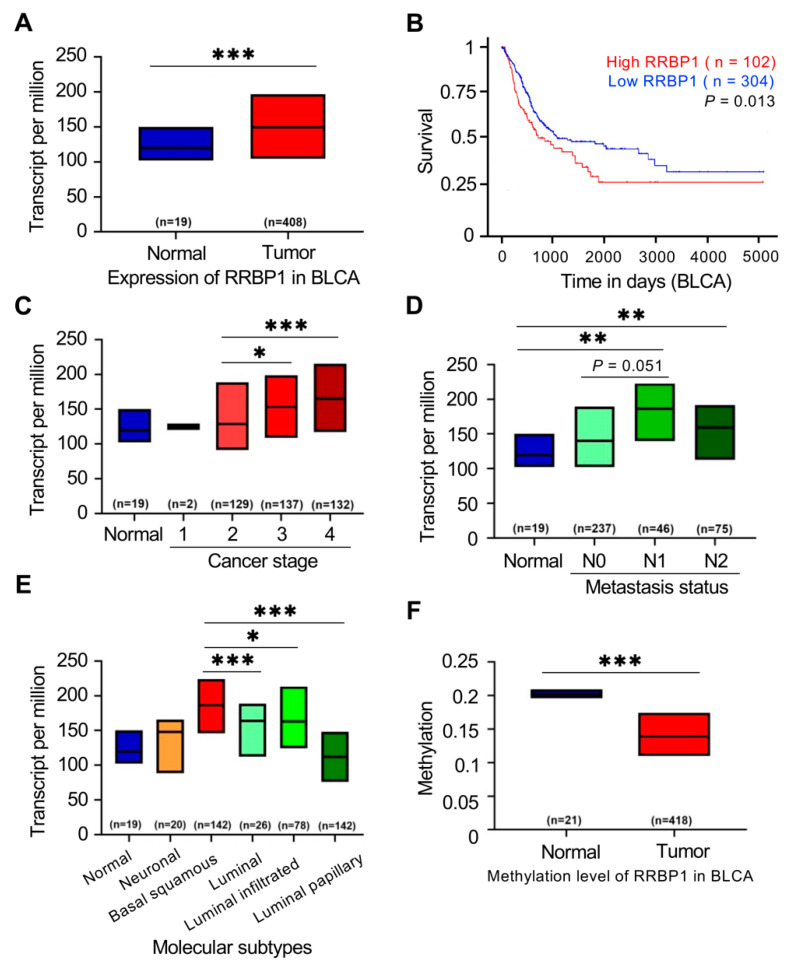
RRBP1 is hypomethylated in BLCA and is correlated with tumor malignancy. (**A**) High expression of RRBP1 transcripts in BLCA tumors. (**B**) High expression of RRBP1 is associated with lower survival rate of BLCA patients (log-rank test, *p* = 0.013). (**C**) High expression of RRBP1 in individual cancer stages of BLCA. (**D**) High expression of RRBP1 in metastatic nodal. (**E**) High expression of RRBP in basal squamous and luminal-infiltrated subtypes. (**F**) Low level of methylation in RRBP1 promoter. (* *p* < 0.05; ** *p* < 0.01; *** *p* < 0.001.) Student *t*-test with two-tailed *p*-value.

To further understand the importance of RRBP1 in clinical prognosis, bladder tumor tissue data were analyzed from TCGA. The expression level of RRBP1 was relatively high in tumor tissues (Figure 2A). Further analysis determined whether expression of RRBP1 was related to the clinical prognosis, with the survival rate of a high level of RRBP1 being significantly lower than that of a low level (Figure 2B).

We further explored whether RRBP1 expression was correlated with cancer stage, nodal metastasis status, and molecular subtypes in bladder cancer. RRBP1 expression was significantly higher in late-stage tumors (stage 3 and stage 4) compared to normal tissues or early stage tumors (*p* < 0.001) (Figure 2C). The expression of RRBP1 in tumor tissues (N0, N1, and N2) was significantly higher than that in normal tissues (*p* < 0.001; Figure 2D). Increased expression of RRBP1 was also observed in tumor tissues with regional lymph node metastasis (N1 and N2) compared to those with no regional lymph node metastasis (N0). Basal squamous subtypes displayed higher RRBP1 expression than the luminal, luminal-infiltrated, and luminal papillary subtypes. There was no statistically significant difference in the neuronal subtypes between the other two groups (Figure 2E). The methylation of the promoter region of the RRBP1 gene in normal tissue and tumor tissue was compared. The methylation of the RRBP1 gene in tumor tissue was low compared to that in normal tissue (Figure 2F). These analyses of TCGA bladder cancer data demonstrated that RRBP1 was an oncogene that was associated with poor prognosis. Expression of RRBP1 was high in tumor tissues with advanced stage and lymphatic metastasis.

### 2.3. Low Methylation of RRBP1 Gene on Chromosome 20: 17,613,678-17,682,283 in UTUC Patients

**Figure 3:**
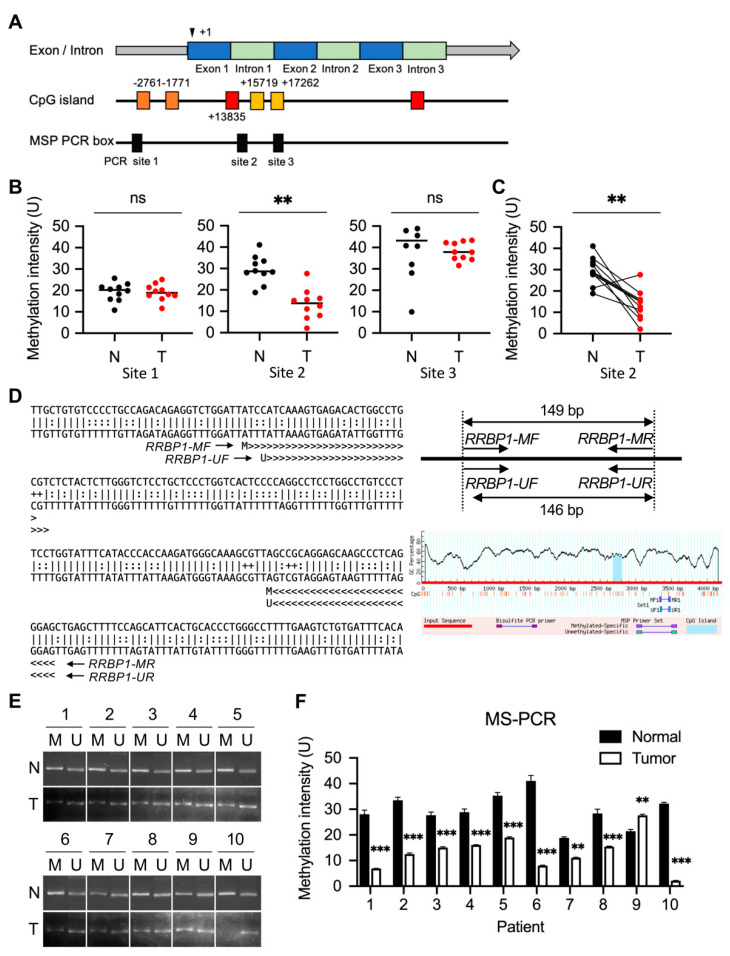
Low methylation of RRBP1 in tumors of UTUC. (**A**) RRBP1 is encoded on chromosome 20. Predicted CpG islands of RRBP1 in the region of exon 1 to intron 3 (chromosome GRCh38:17,613,678-17,682,283) are represented. Illustration represents the design of the methylation-specific PCR assay and the position of the primer sets in CpG islands. (**B**) Low methylation in CpG island of RRBP1 in N/T pairs of UTUC tumors. (** *p* < 0.01.) Student *t*-test with two-tailed *p*-value. (**C**) Methylation intensity of RRBP1 in N/T pairs of UTUC tumors. (** *p* < 0.01.) Paired *t*-test with two-tailed *p*-value. (**D**) Sequence of predicted primer sets for site 2 CpG island. (**E**) Differential level of methylation in site 2 CpG islands of RRBP1 from N/T pairs of UTUC. (**F**) Quantification of methylation in site 2 CpG islands of RRBP1 from N/T pairs of UTUC. (** *p* < 0.01; *** *p* < 0.001.) Statistical analysis was performed by using two-way ANOVA with Tukey’s multiple comparison test.

A negative correlation was evident between gene methylation and its expression in RRBP1. The extent of RRBP1 methylation in patients with UTUC tumors was further analyzed by utilizing the database of CpG islands and analytical tools (DBCAT) and MethPrimer software to predict the CpG islands of the RRBP1 genome. Six CpG islands were predicted (website: http://dbcat.cgm.ntu.edu.tw, accessed on 10 May 2021 and https://www.urogene.org/methprimer/, accessed on 10 May 2021). Three methylation-specific (MS)-PCR boxes were identified, and the sequences of primer sets were designed (Figure 3A). An MS-PCR assay was performed to determine the level of RRBP1 methylation at sites 1, 2, and 3 of the MS-PCR boxes (*p* = 0.9929, 0.0014, and 0.7357, respectively) in 10 patients. Methylation of RRBP1 was low in tumors compared to normal samples at site 2 (Figure 3B). The methylation intensity of N/T pairs at site 2 revealed a significant reduction in RRBP1 (Figure 3C). The sequence and primer sets were designed using MethPrimer software (Figure 3D). The DNA agarose gel electrophoresis quantification of RRBP1 methylation was performed (Figure 3E,F) and the N/T pairs of RRBP1 methylation in sites 1 and 3 of the MS-PCR boxes were quantified by DNA agarose gel electrophoresis (Supplementary Figure S1). In UTUC, low methylation in the RRBP1 genome was negatively correlated with the expression of RRBP1. To further determine the methylation status of RRBP1 in cell lines, we used MS-PCR to detect the methylation levels in SV-HUC-1, RT4, T24, J82, and BFTC909 cells. After the chromosomal DNA of the cells was extracted, RRBP1 methylation was detected by MS-PCR using site 2 of the MS-PCR box shown in Figure 3B. Higher RRBP1 methylation in the normal cell line of urothelial nontumor tissue SV-HUC-1 was determined using DNA agarose gel analysis. RRBP1 methylation in RT4 (low-metastatic cancer cell line), T24 and J82 (advanced bladder cancer cell lines), and BFTC909 (advanced urothelial cancer cell line) were lower than that in the normal cell line (methylation intensity: 36.42 unit for SV-HUC-1 and 28.32, 12.78, 11.56, and 17.65 for RT4, T24, J82, and BFTC909, respectively) (Supplementary Figure S2).

### 2.4. RRBP1 Is Highly Expressed in UTUC Tumor Tissues and Cancer Cell Lines of Urothelial Tumors

**Figure 4:**
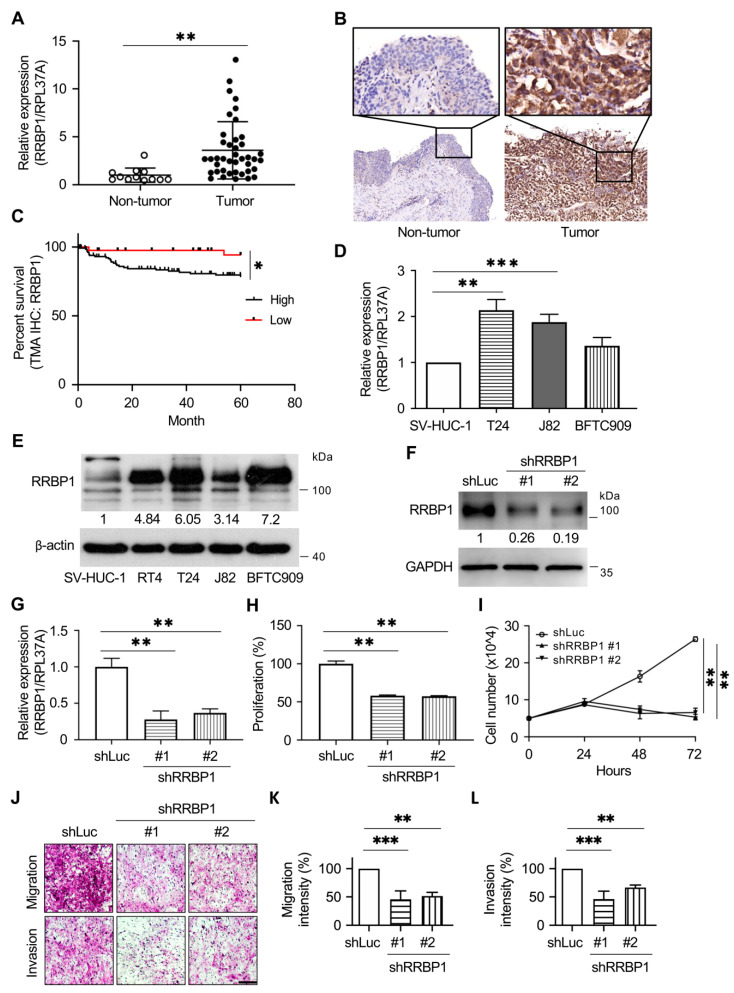
High expression of RRBP1 in tumors from patients with UTUC. (**A**) Levels of RRBP1 mRNA are higher than nontumor in UTUC. The mRNA expression of RRBP1 was analyzed based on the unlinked database from National Health Institute. (** *p* < 0.01.) Student *t*-test with two-tailed *p*-value. (**B**) High expression of RRBP1 in tissues from UTUC tumors. Tissues of nontumor and tumor were fixed and embedded in paraffin. Immunohistochemistry staining of tissue sections was performed with RRBP1 antibody. (**C**) Analysis of survival within five years according to the immunohistochemistry staining in tumor tissue microarray (*n* = 197). (* *p* < 0.05.) Log-rank test. (**D**) Relative expression of RRBP1 in cell lines. mRNA of SV-HUV1, J82, T24, and BFTC909 cells were collected for quantitative PCR assay targeting RRBP1 (** *p* < 0.01; *** *p* < 0.001). (**E**) Protein expression of RRBP1 in cell lines. Cell lysates were harvested and protein expression was detected by Western blotting assay with RRBP1 antibody. (**F**) Silencing of RRBP1 in BFTC909 cell. BFTC909 cells were transfected with shRNA for RRBP1#1 and RRBP1#2. (**G**) The mRNA was collected for quantitative PCR analysis targeting RRBP1 (RPL37A: internal control) (** *p* < 0.01). (**H**,**I**) Silencing of RRBP1 reduced cell proliferation. BFTC909-shLuc, shRRBP1#1, and shRRBP1#2 cells were seeded in a 96-well plate and cultured for 24, 48, and 72 h. The cell number was counted or determined by the WST-1 assay (** *p* < 0.01). (**J**–**L**) Silencing of RRBP1 reduced cell migration and invasion. BFTC909-shLuc, shRRBP1#1, and shRRBP1#2 cells were seeded in the Transwell inserts, and cell migration or invasion was performed with incubation at 37 °C for 18 h. The crystal-violet-stained cells were lyzed and analyzed (** *p* < 0.01; *** *p* < 0.001.). Statistical analysis was performed by using one-way ANOVA with Dunnett’s test. Scale bar = 100 µm.

As shown in Figure 3F, low methylation in the RRBP1 genome was found in the tumor samples of UTUC. There were significant differences between tumor and nontumor tissues. Real-time PCR analysis showed that the expression of RRBP1 mRNA in tumor sites was lower than that in nontumor sites (Figure 4A). Expression of RRBP1 in tumor samples was further analyzed, with protein expression detected by immunohistochemistry (IHC). The analysis revealed relatively high expression of RRBP1 protein compared to the expression in nontumor samples (Figure 4B).

All the above results echoed TCGA findings that RRBP1 expression was increased in bladder cancer tumors compared to normal urothelial tissues. The same findings were obtained with UTUC tumors. Tissue microarray was further used for immunostaining and statistical analysis in 197 UTUC patients. The results showed that the expression of RRBP1 was significantly correlated with the incidence of distant metastasis and low survival within 5 years, indicating that higher RRBP1 predicted higher occurrence of distant metastasis and poor survival (Figure 4C and Supplementary Table S1).

In addition, we examined whether a similar phenomenon could be observed in human urothelial cancer cell lines. Real-time PCR was used to analyze RRBP1 mRNA expression. The analysis revealed a low level of RRBP1 mRNA expression in SV-HUC-1 cells, with relatively high expression in J82 and T24 cells (Figure 4D). RRBP1 protein expression in SV-HUC-1 cells was significantly lower than the expression in RT4, J82, T24, and BFTC909 cells in a Western blot assay (Figure 4E and Figure S3). Short hairpin (sh) RRBP1 purchased from the RNA interference (RNAi) core facility in Taiwan was used to inhibit the expression of RRBP1 in BFTC909 UTUC cells. Forty-eight hours after transfection of the shRRBP1 plasmid targeting BFTC909, both mRNA and protein expression levels were inhibited (Figure 4F). RRBP1 was previously associated with cell proliferation [35]. In the present study, WST-1 analysis performed 48 h after transfection of shRRBP1 with BFTC909 showed that RRBP1 was inhibited and cell proliferation was reduced (Figure 4H). Cells were counted at 24, 48, and 72 h after shRRBP1 transfection. The results confirmed that inhibition of RRBP1 reduced cell proliferation (Figure 4I).

As the expression of RRBP1 is related to migration and invasion in bladder cancer [36], we next determined whether the elimination of RRBP1 in the cell line inhibited cell mobility. We used a Boyden chamber with and without Matrigel coating for the migration and invasion assays, respectively. BFTC909-shLuc, -shRRBP1#1, and #2 cells were seeded in the upper chamber for 24 h, and cell migration was assessed. After 18 h of migration, the cells on the lower side of the membrane were fixed and stained with crystal violet. Because the cell number of migrated cells in BFTC909 was difficult to count, the crystal-violet-stained cells were quantitatively analyzed and modified according to the study of Cvetanova et al., as described in the Materials and Methods section. Silencing of RRBP1 in BFTC909 decreased cell migration and invasion in shRRBP1#1 and #2 cells (migration intensity: 45.76% and 51.43% in BFTC909-shRRBP1#1 and #2 cells; invasion intensity: 46.3% and 66.54% in BFTC909-shRRBP1#1 and #2 cells) (Figure 4J–L).

### 2.5. Analysis of Molecular Subtypes of RRBP1-Correlated Gene Expression in UTUC

**Figure 5:**
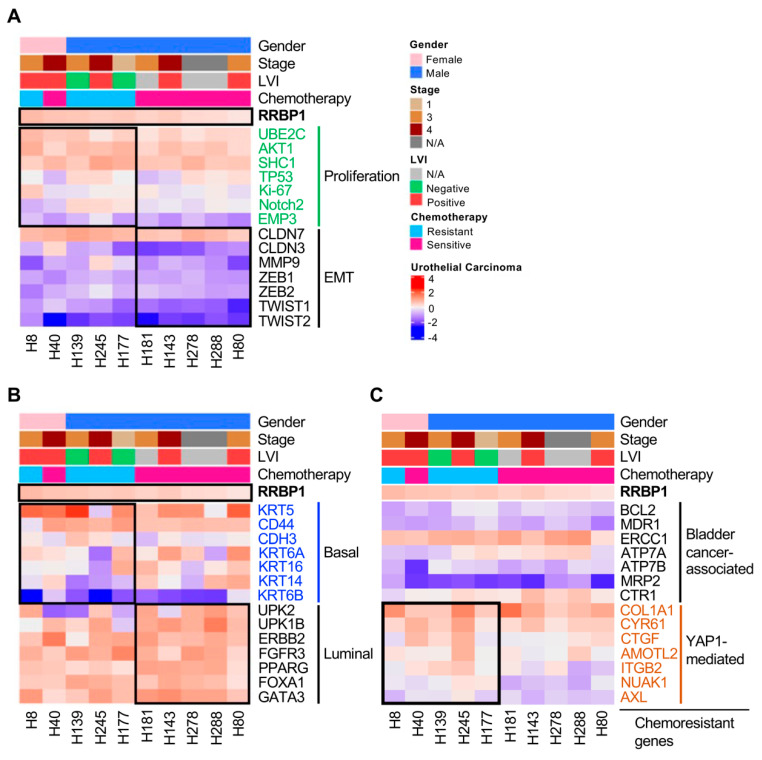
Analysis of mRNA expression of molecular subtypes according to the level of RRBP1 in UTUC. Heatmap analysis of mRNA expression represented a high correlation between RRBP1 and chemotherapy. RNA expression of molecular subtypes was analyzed according to the level of RRBP1. Ten samples were analyzed using the unlinked database from the National Health Institute. (**A**) Clustering of genes for the proliferation and EMT biomarkers. (**B**) Clustering of genes for the basal and luminal biomarkers. (**C**) Clustering of genes for bladder-cancer-associated and YAP1-mediated chemoresistant genes.

Using the tissue bank database from the NIH, RNA sequencing results for 10 samples were analyzed. The clinical data were classified according to sex, stage, confirmed lymphovascular invasion (LVI), and chemotherapy efficacy. Ranking of RRBP1 expression according to high to low RRBP1 expression revealed that RRBP1 expression was not correlated with sex, tumor stage, and LVI, but was related to the efficacy of chemotherapy drugs. The higher the RRBP1 expression, the worse the response to chemotherapeutic agents (Figure 5A). Previous studies have indicated that RRBP1 might have an effect on drug resistance [26]. Proliferation markers were then examined. Patients with high RRBP1 expression had higher expression of UBE2C, Akt1, TP53, Notch2, and EMP3, which promote cell proliferation (Figure 5A). These findings confirmed that RRBP1 reduction inhibits cell proliferation, as shown in Figure 4F,G. Further analysis of epithelial–mesenchymal transition (EMT)-related genes showed that patients with low RRBP1 expression displayed relatively low gene expression levels of CLDN7, CLDN3, MMP9, ZEB2, and Twist1, which are related to metastasis (Figure 5B). These findings also show the phenomenon of nodal metastasis in bladder cancer data that was evident in the analysis of TCGA data (Figure 2D). High RRBP1 expression is associated with tumor invasion and metastasis. We observed that basal squamous subtypes had higher RRBP1 expression compared to luminal, luminal-infiltrated, and luminal papillary subtypes (Figure 2E). Thus, the distribution of related genes in UTUC was analyzed using basal cell subtype markers and luminal markers. In patients with high RRBP1 expression, the expression levels of basal-marker-related genes, including KRT5, CD44, CDH3, and KRT6a, were increased. Conversely, patients with low RRBP1 expression levels displayed increased expression levels of luminal-marker-related genes, including UPK2, UPK1B, ERBB2, FGFR3, PPARG, FoxA1, and GATA3 (Figure 5B). When RRBP1 expression was increased, the expression of basal markers was correspondingly increased and the expression of luminal markers was decreased.

We further analyzed the association between RRBP1 and chemoresistant genes according to two important studies: (1) Mari et al. identified chemoresistance genes in bladder cancer [37]; and, recently, (2) Shriwas et al. found that RRBP1 contributes to the expression of YAP1 and influences cisplatin-based chemotherapy through the regulation of YAP1-mediated genes [38]. We analyzed the relationship between RRBP1 and genes involved in bladder-cancer-associated or YAP1-mediated chemoresistance and genes. There was not much difference in the correlation between RRBP1 expression and bladder-cancer-associated drug-resistance genes reported by Andrea et al. (Figure 5C). Interestingly, RRBP1 and chemoresistance were highly correlated with YAP1-targeted genes. Our results showed that RRBP1 levels were positively correlated with YAP1 involved chemoresistance genes (COL1A1, CYR61, CTGF, AMOTL2, ITGB2, NUAK1, and AXL), which is consistent with the findings of Shriwas et al.

### 2.6. Tumors with Low Expression of RRBP1 Are Sensitive to Cisplatin, Gemcitabine, and Epirubicin

**Figure 6:**
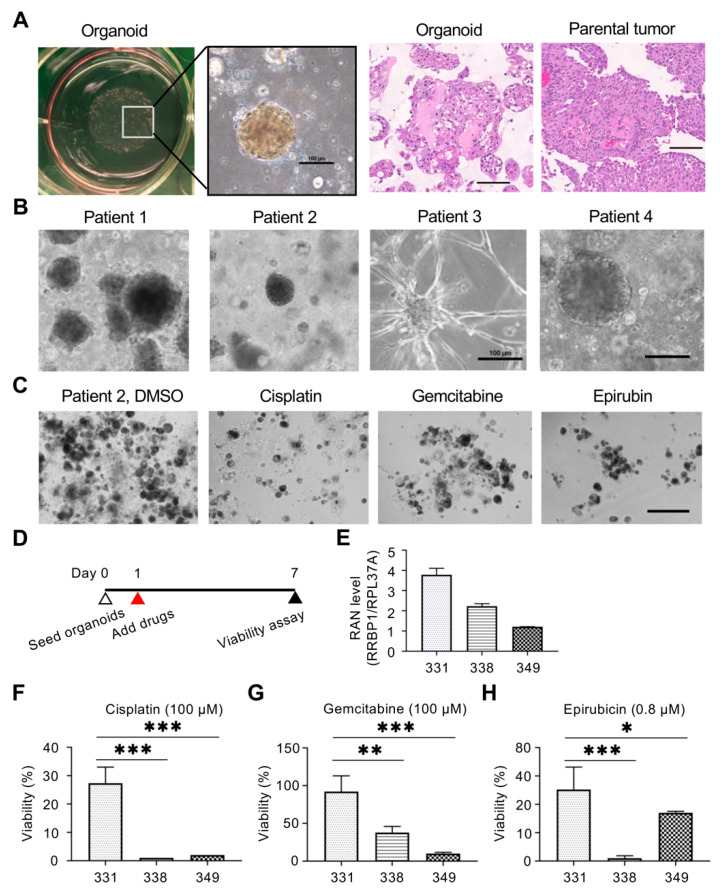
RRBP1 shows potential for drug combination therapy. (**A**) UTUC patient-derived organoid culture system mimics the cell morphology similar to the parental tumor. The cells were extracted from the patient’s tumor and then embedded in Matrigel to establish organoid cultures. HE staining was used to detect sections from the parental tumor and organoid. Scale bar = 100 µm. (**B**) Representative images for the four primary UTUC organoids. The organoids were constructed from the patient’s tumor and cultured for two weeks. Scale bar = 100 µm. (**C**) The organoids were treated with 100 µM drugs for 7 days. Scale bar = 100 µm. (**D**) The organoids were seeded overnight and then treated with drugs for 7 days. The viability of organoids was determined by the 3D organoid viability assay. (**E**) The levels of RRBP1 mRNA in three organoids were measured. Data represent the mean ± SD. (**F**–**H**) The level of RRBP1 mRNA was correlated to the cytotoxicity effect of drugs in organoids. The organoids were treated with 100 µM cisplatin, gemcitabine, and 0.8 µM epirubicin for 7 days, and then the viability was determined. Data represent the mean ± SD. (* *p* < 0.05; ** *p* < 0.01; *** *p* < 0.001.) Statistical analysis was performed by using one-way ANOVA with Dunnett’s test.

Since the expression of RRBP1 might be related to chemotherapy with drug treatment (Figure 5A), the PDO model was used to determine whether RRBP1 was a potential target for enhancing chemotherapy. Tumor organoids obtained from patients were cultured on plates containing Matrigel, and then observed by bright field microscopy (Figure 6A). Observation of tumor sections revealed that the tumor organoids were similar in cell morphology and retained the characteristically pronounced tumor heterogeneity (Figure 6A).

Organoids from four patients were collected and cultured, and their growth was observed by microscopy. Each tumor organoid differed in morphology and tumor sizes from the same patient varied, revealing that these organoids retained high heterogeneity, as per the original tumors (Figure 6B). The drug effect was then tested by treatment of tumor organoids with 100 μM cisplatin, gemcitabine, and epirubicin, and microscopy observation 7 days later (Figure 6C). The effect of restriction on the growth of organoids after treatment was obvious for the different drugs, with the titrated tumor organoid samples then embedded into 96 wells containing Matrigel, and chemotherapeutic agents administered the next day. Cell survival was analyzed on day 7 (Figure 6D).

The effects of the drugs on the growth inhibition of tumor organoids were assessed by determining the half-growth-limiting dose (IC50) of cisplatin (1.812–3.478 µM), gemcitabine (1.817 µM), and epirubicin (0.258–1.066 µM) (Supplementary Figure S4). mRNA expressions of RRBP1 were analyzed in patients 331, 338, and 349. Patient 331 displayed the highest RRBP1 mRNA expression (4-fold greater than the expression by patient 349). The expression by patient 338 was 2.3-fold higher than the expression by patient 349 (Figure 6E). A drug sensitivity assay was performed in different tumor organoids at a growth-limiting dose of IC30 for patients 331 and 338, and the survival rates of tumor organs were determined after 7 days. After treatment with 100 μM cisplatin, cell viability of patient 331 was significantly higher than that of patients 338 and 349 (27.6%, 0.6%, and 1.8%, respectively). The *p*-values of 331/338 and 331/349 were 0.0002, respectively (Figure 6F). After treatment with 100 μM gemcitabine, cell viability of 331 was significantly higher than that of 338 and 349 (91.2%, 38.9%, and 11.4%, respectively (331/338, *p* = 0.0045; 331/349, *p* = 0.0008; Figure 6G)). After treatment with 0.8 μM epirubicin, cell viability of 331 was significantly higher than that of 338 (49.1% and 0.9 %) (331/338, *p* = 0.0017; 331/349, *p* = 0.1508; Figure 6H). Interestingly, organoids with higher RRBP1 expression showed a poorer response to chemotherapy.

The collective findings demonstrated that the expression of RRBP1 was significantly higher than that of para-tumors in clinical tissue staining. Tumor organoids were similar in morphology, and hematoxylin and eosin staining of the original tumor sections. High expression of the RRBP1 organoid was associated with low drug sensitivity. Tumor organoids with low RRBP1 expression appeared to be more chemosensitive.

## 3. Discussion

The ER is important for the synthesis, modification, folding, and secretion of proteins, which affects cellular function and survival. ER abnormalities often play a carcinogenic role in tumor growth and in the tumor microenvironment. Abnormal activation of ER proteins and their downstream signaling pathways have become key therapeutic targets for tumor growth and metastasis [39]. RRBP1 is crucial in ribosomal binding and the termination of differentiation in secretory cells and tissues in the ER [20,21,22]. In this study, the upregulation of RRBP1 in human UTUC was associated with malignant stage, metastasis, and poor prognosis of the tumors (Figure 2). CpG island methylation of RRBP1 in cancer cell lines was also confirmed, and RRBP1 was found to be hypomethylated in tumor cells (Figure 4). The examination of clinical samples also revealed the hypomethylation of RRBP1 in UTUC tumor tissues, while tumor cells that highly expressed RRBP1 displayed a high tolerance to clinical drugs. These findings could provide a reference for treatment strategies for tumors affected by relevant mechanisms (Figure 5 and Figure 6).

The upregulated expression of RRBP1 in lung cancer, colorectal cancer, endometrial endometrioid adenocarcinoma, ovarian, breast, and bladder cancer leads to poor prognosis [22,26,29,30,35,40]. The inhibited expression of RRBP1 could potentially reduce the proliferation and metastasis of cancer cell lines. The RRBP1 gene has a low mutation rate in tumors, with no missense or nonsense mutations detected in patients, indicating that its expression in tumors affects the activity of cancer cells, rather than through mutations or deletions. Our real-time qPCR and Western blot analysis were performed to analyze RRBP1 in cell lines, and inconsistent mRNA and protein expression were observed in J82 and BFTC909 in Figure 4E,F. Similar results were also found in some lung cancer cell lines [26], suggesting that this protein may have a post-translational modification mechanism. In J82 cells, RRBP1 is likely to undergo a rapid protein turnover mechanism, and this process may be related to the protein ubiquitination [41]. It is speculated that the abnormal post-translational modification and protein turnover of RRBP1 may be related to malignant tumor formation. Tsai et al. reported that inhibition of RRBP1 reduced GRP78 expression and promoted tunicamycin-induced apoptotic cell death in human non-small-cell lung cancer [26]. He et al. reported that, in hepatocellular carcinoma, high glucose levels could promote the proliferation and metastasis of cancer cells by upregulating the E2F/RRBP1 pathway [42]. UBE2C, AKT1, and SHC1 expressions were upregulated in RRBP1-mediated UTUC gene proliferation. The expressions of CLDN7, CLDN3, MMP9, and Twist1 were upregulated in the RRBP1-mediated EMT of UTUC (Figure 5). Among these, UBE2C has been demonstrated to be a high tumor marker associated with advanced cancer. In malignant glioma, breast cancer, gastric cancer, melanoma, endometrial cancer, head and neck squamous cell carcinoma, and oral squamous cell carcinoma (OSCC), UBE2C is associated with poor prognosis [43,44,45,46,47,48,49,50]. In EMT-related genes, Claudin-7 (CLDN7) promotes EMT in colon cancer and ovarian cancer enhanced invasion [51,52]. In ovarian cancer, CLDN3 increases metastasis [53].

The endoplasmic reticulum (ER) restores proteostasis through folding and post-translational processing of membrane-bound and secreted proteins. Misfolded proteins are the target of proteolysis, and external stimuli, such as nutritional deprivation, hypoxia, acidosis, drug toxicity, and radiation, can easily lead to the accumulation of misfolded proteins under ER stress conditions [54,55]. To maintain ER homeostasis, a process called the unfolded protein reaction (UPR) slows down ongoing protein synthesis and increases the folding capacity of the ER. If this adaptive response does not restore protease homeostasis, the proapoptotic proteins of the Bcl-2 family (BH3-only proteins) are activated, eventually leading to programed cell death [56]. Glucose regulatory protein 78 (GRP78) and binding immunoglobulin protein (BiP) maintain ER homeostasis. During ER stress, BiP is separated from three different transmembrane sensor-controlling proteins: inositol requires enzyme 1α (IRE1α), protein kinase RNA (PKR)-like ER kinase (PERK), and activating transcription factor 6 (ATF6) [56]. They are induced to activate downstream pathways to promote protein folding [57]. If the different UPR-mediated mechanisms fail to counteract ER stress, apoptotic pathways are activated [57].

RRBP1 promotes UPR during ER stress. It regulates UPR via GRP78, thereby disrupting ER stress induced by tunicamycin and 2-deoxy-D-glucose [58]. Thus, RRBP1 may play a key role in maintaining tumor cell survival under stress conditions. ER stress has anti- and protumor effects on tumor development. It can promote the autophagy’s protective function in cells by prolonging UPR activation, thus leading to the mechanism that triggers cell death [57]. However, cancer cells can also use UPR as an adaptive mechanism to support tumor cell survival and chemotherapeutic resistance [59].

In our study, RRBP1 was negatively correlated with the outcome of chemotherapy in some patients, and it was speculated that, in patients with low RRBP1, the UPR mechanism was disrupted, thus promoting the chemotherapeutic effect. Therefore, it is speculated that the use of ER-induced drugs may also destroy the UPR adaptive system and achieve antitumor effects in these patients. UPR regulates autophagy by inhibiting the activity of mTOR, a negative regulator of autophagy, leading to the accumulation of autophagosomes in a beclin-1- and Atg-7-dependent manner [60]. Therefore, it is speculated that RRBP1 may promote tumor growth and antichemotherapy drugs by regulating UPR-mediated autophagy during ER stress. Further experiments are required to confirm this.

Van de Wetering et al. used colonic organoids to confirm that the molecular profile was consistent with that of the original tumor [61]. Lee et al. found that the use of patient-derived bladder organoids could also reproduce the high features of the original tumor and could be applied to histopathological and molecular subtype analyses of both non-muscle-invasive and muscle-invasive features [32]. Referring to the method of the latter authors, this study established tumor-like organoids of UTUC and showed that they could represent the complete characteristics of human UTUC. The tumor-like organoids displayed the tumor diversity and significantly preserved the heterogeneity, growth characteristics, and morphology of tumors [62]. PDOs could provide a platform for precise cancer medical testing, including validation of mutation points and characterization, high-throughput screening of therapeutic drugs, and a reference for individual treatment strategies. Chemotherapy drugs screened by organoids can then be validated in mouse models to establish a platform for organoid screening in mouse models [63,64]. PDOs could be a potential platform for prediction of chemosensitivity, the effect of radiation, and response to immune checkpoint inhibitors [65,66,67], and so could be a focus of future research in combining clinical studies to confirm whether the effect of PDOs on drug treatment could reflect the effect of tumor treatment in patients. Our clinical cohort to validate the relationship between RRBP1 and prognosis was localized to UTUC without the use of NAC. In the future, the role of RRBP1 and its relationship with chemotherapy response in a real-world chemotherapy-based cohort should be investigated based on the present PDO findings.

Recent studies have shown that the level of RRBP1 is increased in many human cancers and is associated with tumorigenesis, metastasis, and poor prognosis. The level of RRBP1 is also related to the signal transduction of cell proliferation regulated by GRP78 or E2F, indicating the importance of RRBP1 as a tumor marker and possibly as a therapeutic target for the inhibition of cancer cell growth [26,39]. The present results showed that RRBP1 was upregulated in UTUC and promoted the growth of cancer cells. Among the genes affected by RRBP1, UBE2C, SHC1, and CLDN7 have all been recently identified as highly relevant targets in other cancers. It was further found that patients with low RRBP1 expression in UTUC samples were more sensitive to cisplatin, gemcitabine, epirubicin, and other drugs, providing a reference for treatment strategies.

## 4. Materials and Methods

### 4.1. Tissue Samples and Cell Lines

Tissue specimens of UTUC were collected from patients at Chang Gung Memorial Hospital in Kaohsiung. The study was approved by the Institutional Review Committee of the Chang Gung Medical Foundation (IRB number: 201504731B0) on 14 September 2015. SV-HUC-1 (CRL-9520, ATCC), J82 (HTB-1, ATCC), BFTC909 (60069, BCRC), and T24 (60062, BCRC) cell lines were purchased from the Biological Resource Collection and Research Center. The plasmids were isolated using a commercial plasmid mini kit (QIAGEN, Valencia, CA, USA) and were used according to the manufacturer’s instructions. In vitro DNA transfection was performed using the PolyJet reagent (SignaGen Laboratories, Frederick, MD, USA).

### 4.2. Methylation-Specific PCR

DNA methylation was performed as described previously [68]. Briefly, chromosome DNA was extracted from 10 normal and 10 tumor tissue samples using the QIAamp DNA Mini Kit (QIAGEN, Hilden, Germany). For methylation-specific PCR, primers were designed using MethPrimer (https://www.urogene.org/methprimer/) on 10 May 2021. The primer sequences are listed in Supplementary Table S2. DNA (500 ng) was converted using the EZ DNA methylation kit based on bisulfite conversion (Zymo Research, Irvine, CA, USA). The bisulfite transformation was performed in the dark at 50 °C for 16 h. The transformed DNA was desulfurized and eluted by adding 20 μL elution buffer. CpG methylation levels were detected by PCR amplification using the HotStarTaq^®^ MasterMix kit (QIAGEN, Hilden, Germany, Cat. No. 203443). The amplification conditions were 40 cycles of 95 °C for 1 min, 9 °C for 30 s, 55–60 °C for 30 s, 72 °C for 30 s. The relative methylation levels of each CpG site were analyzed using ImageJ software (version 1.8; NIH, Bethesda, MD, USA).

### 4.3. Analysis of Clinical Characteristic and Methylation Status of RRBP1 in the BLCA Database

UALCAN (http://ualcan.path.uab.edu/index.html) was used to detect RRBP1 mRNA expression and promoter methylation in the characteristics of each patient in the TCGA dataset on 10 May 2021. Data from probes cg02460349, cg07597892, cg12212206, cg03001504, cg26447697, and cg03704771 in Infinium HumanMethylation450K BeadChips (Illumina, San Diego, CA, USA) were analyzed for promoter methylation data. The plots and labels downloaded from UALCAN were modified for readability.

### 4.4. Immunohistochemistry and Patient Grouping

Human UTUC tissue microarray contained 197 tumor samples with triplicate cores for each sample. All samples were obtained from the tissue bank of Chang Gung Memorial Hospital, Kaohsiung, Chang Gung Medical Foundation. The tissue samples were fixed with formalin, embedded in paraffin, and sectioned. Tissue immunostaining was performed automatically using a fully automated Bond-Max instrument (Leica Microsystems, Wetzlar, Germany) according to the manufacturer’s instructions. The settings for the steps were as follows: (1) dewaxing: rinsing of the glass slides with dewaxing solution at 72 °C; (2) antigen retrieval: immersion of tissue slides in antigen retrieval buffer at 100 °C for 10 min; (3) peroxide block: immersion of glass slides in hydrogen peroxide solution and reaction at room temperature for 10 min; (4) primary antibody reaction: RRBP1 (1:100; ab95983, Abcam) at RT maintained for 60 min; (5) post-primary reagent reaction at room temperature for 10 min; (6) staining with 3,3′-diaminobenzidine tetrahydrochloride (DAB) at room temperature for 3 min; and (7) counterstaining with hematoxylin stain for 1 min. After mounting the tissue slides, a Vectra Polaris Automated Quantitative Pathology Imaging System was used to scan the slides (PerkinElmer, Boston, MA, USA). The scoring of RRBP1 in the IHC assay was referenced in our previous publication [34]. The immunoreactivity scoring was based on the intensity of positive staining using a three-point scale: 0–10%, 0; 11–50%, 1; 51–80%, 2; and >80%, 3. Tumor morphology and RRBP1 levels on the slides were also verified by a urological pathologist (Dr. Min-Tse Sung) and urological oncologist (Dr. Hao-Lun Luo) in our hospital.

#### 4.5. shRNA, PCR, and Quantitative Real-Time PCR

RNA was knocked down using virus harboring the appropriate shRNA, obtained from the RNAi Core (Academic Sinica). The shRNA targeting sequences of RRBP1 were shRRBP1#1: GTGAAGCATCTCGAAGAGATT and shRRBNP1#2: CAGGCAGCAGTTGAGTGAAAT. PCR was performed using PFU Turbo polymerase (Agilent) according to the manufacturer’s instructions. qRT-PCR was performed using SYBR Green PCR Master Mix (Thermo Fisher Scientific, Waltham, MA, USA) and ABI StepOnePlus sequence detection system (Thermo Fisher Scientific, Waltham, MA, USA). The real-time PCR primers were as follows: RRBP1 forward, 5′-TCCTGTCTGAGAAGGCTGGCAT-3′; RRBP1 reverse primer, 5′-CCTCAGTTTGCTCTTGGCGACA-3′; RPL37A forward, 5′-AATCAGCCAGCA CGCCAAGTAC-3′; RPL37A reverse primer, 5′-GCCACTGTCTTCATGCAGGAAC-3′.

### 4.6. Western Blotting

Cell lysates were extracted using RIPA buffer and quantified using a BCA protein analysis kit (Pierce, Rockford, IL, USA). Twenty micrograms of lysate was loaded onto a polyacrylamide gel and SDS-PAGE was performed. The separated proteins were transferred to an Immobilon-P transfer polyvinylidene fluoride membrane (Millipore, Billerica, MA, USA). Each membrane was incubated with primary antibody to RRBP1 (ab95983; 1:1000 dilution; Abcam) and glyceraldehyde 3-phosphate dehydrogenase (MAB374; 1:1000 dilution; Millipore) overnight and for 2 h, respectively. After reaction with horseradish peroxidase-conjugated secondary antibody (1:2000 dilution; Cell Signaling Technology, Beverly, MA, USA) for 1 h, each membrane was scanned using a UVP ChemStudio PLUS instrument (UVP Inc., Upland, CA, USA) and analyzed with the ImageJ software (version 1.8).

### 4.7. In Vitro Migration and Invasion Assay

Cell migration was measured using Transwell inserts 6.5 mm in diameter with a pore size of 8 μm (Jet Biofil, Guangzhou, China), and the invasion assay was performed using the upper chamber coated with Matrigel (dilution 1:6 in culture media, #356234, Corning, Bedford, MA, USA). Cells were seeded in the upper chambers with 300 μL complete medium and allowed to settle overnight at 37 °C in an incubator with a humidified atmosphere of 5% CO_2_. The same number of cells was seeded to a 96-well plate for cell number normalization using the WST-1 assay (Roche, Mannheim, Germany). The next day, the medium in the upper chamber was changed without serum. The Transwell insert was placed at a lower chamber which was filled with 600 μL serum-containing medium (10% FBS). Cell migration or invasion was performed with incubation at 37 °C for 18 h, and then the Transwell inserts were fixed and the upper side with unmigrated cells was wiped with cotton swabs. The underside of the inserts was stained with 0.1 mg/mL crystal violet and the images were photographed under an inverted microscope (IX51, Olympus, Japan). The crystal-violet-stained cells were quantitatively analyzed and modified according to the study of Biljana et al. [69]. The cells were dissolved in 250 µL 20% acetic acid and the absorbance (O.D. 595 nm) was measured using an ELISA reader (Varioskan LUX Multimode Microplate Reader, Thermo Fisher Scientific, USA). Intensity was first normalized to the cell number of each sample from the WST-1 assay to determine the normalized OD595. Migration/invasion intensity (%) = normalized OD595 (shRNA)/normalized OD595 (shLuc) × 100. The plots of migration and invasion were analyzed using the GraphPad Prism 8 Software (GraphPad, San Diego, CA, USA).

### 4.8. Drug Sensitivity Assay in Organoids

Human organoids were collected after 5 days of culture and the drug response analysis was performed. After digesting the gel with 1 mg/mL dispase at 37 °C for 60 min, the large organoids were removed using a 100 µm cell strainer. Organoids were suspended in 2% matrix/organoid culture medium (150–200,000 organoids/mL) in ultra-low 96-well U-plates in triplicate. After 24 h, a compound was serially diluted at concentrations ranging from 100 mM to 1.28 nM, with dimethylsulfoxide as a control. Cell activity was detected using the Celltiter-Glo 3D Kit (Promega, Madison, WI, USA) and analyzed statistically using GraphPad Prism 8 software (GraphPad, San Diego, CA, USA) after 6 days of drug treatment.

### 4.9. Statistical Analysis

Unless otherwise stated, all in vitro experiments were conducted in at least three separate experiments. Data from the methylation-specific PCR assay, proliferation assay, real-time PCR analyses, and drug sensitivity assays in this study are expressed as mean ± SD. Statistical significance between different experimental groups was analyzed using the Student’s *t*-test (two-tailed). Statistical significance was set at *p* < 0.05. Statistical analyses were performed using GraphPad Prism 8 (GraphPad, San Diego, CA, USA).

## Data Availability

The data presented in this study are available on request from the corresponding author. The data are not publicly available due to privacy.

## Supplementary Materials

The following are available online at https://www.mdpi.com/article/10.3390/ijms22168761/s1.
