# Supplementary material for: Hypomethylated RRBP1 Potentiates Tumor Malignancy and Chemoresistance in Upper Tract Urothelial Carcinoma
Source: Int J Mol Sci. 2021 Aug 16;22(16):8761. doi: 10.3390/ijms22168761 (PMC8395942; doi:10.3390/ijms22168761)
Supplement: Supplementary file 1 [file ijms-22-08761-s001.zip › ijms-1326223-supplementary.pdf]

Supplementary Figure S1

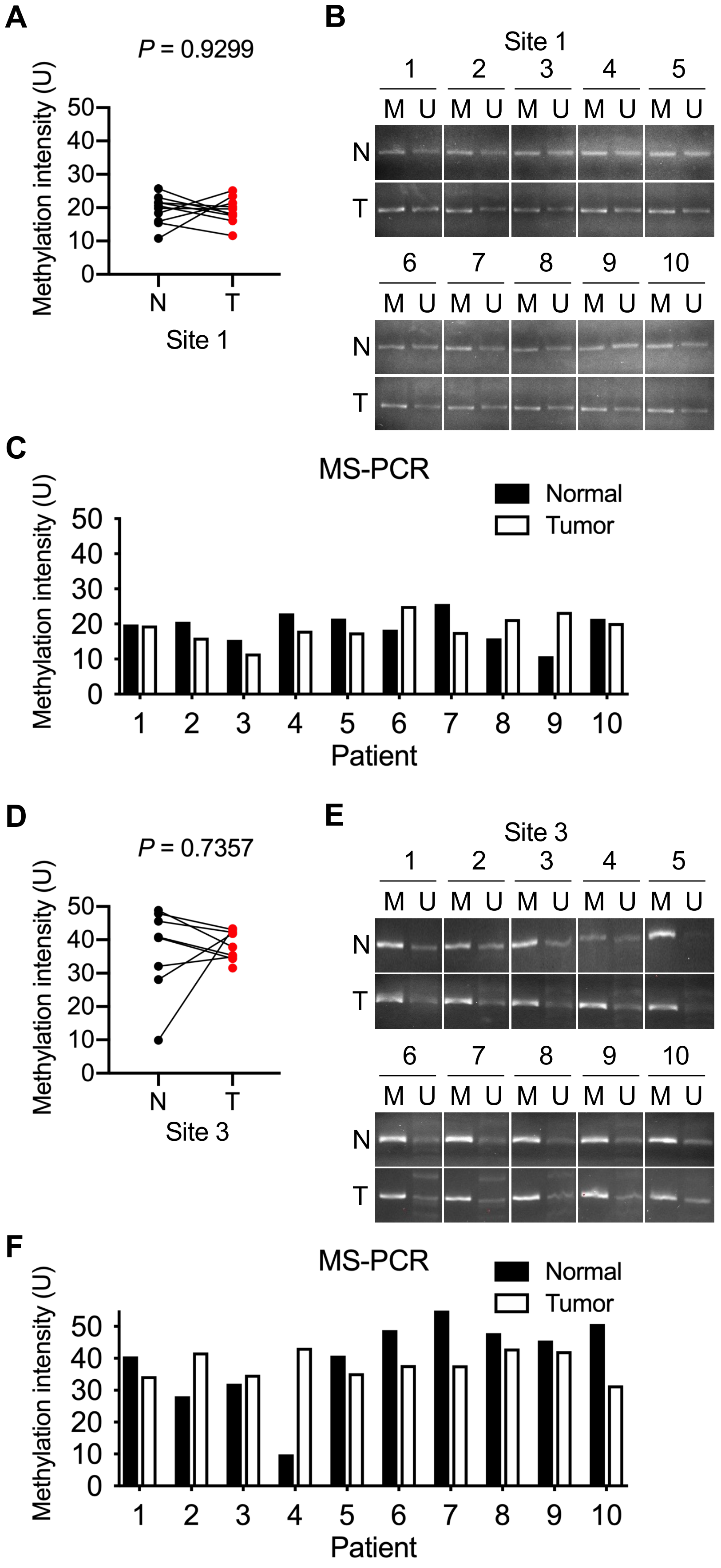

Supplementary Figure S2

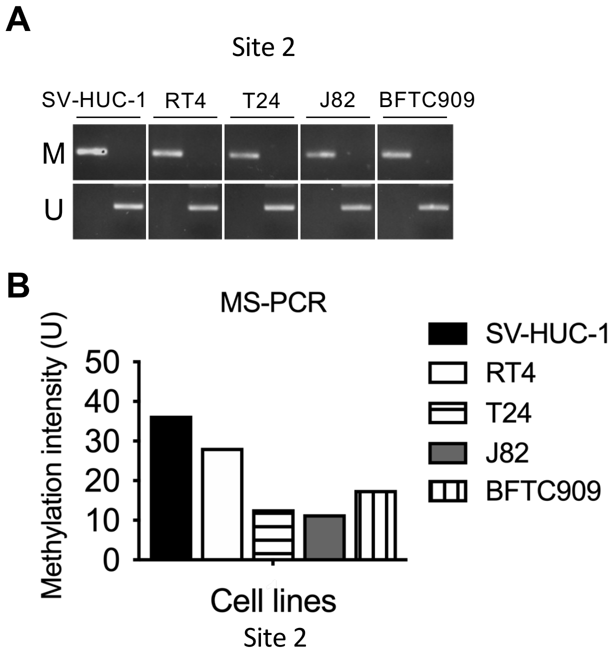

Supplementary Figure S3

A

(Figure 4E, RRBP1)

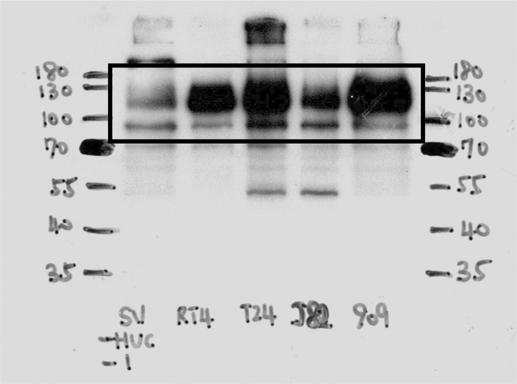

B

(Figure 4E, β-actin)

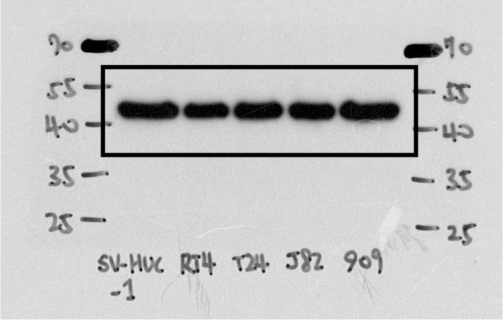

C

(Figure 4F, RRBP1)

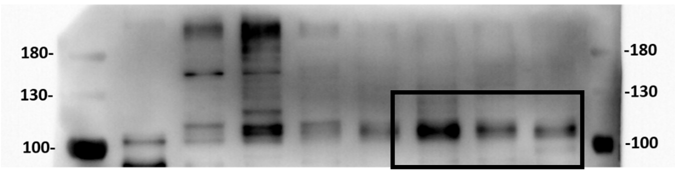

D

(Figure 4F, β-actin)

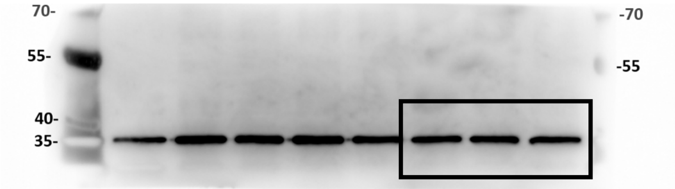

Supplementary Figure S4

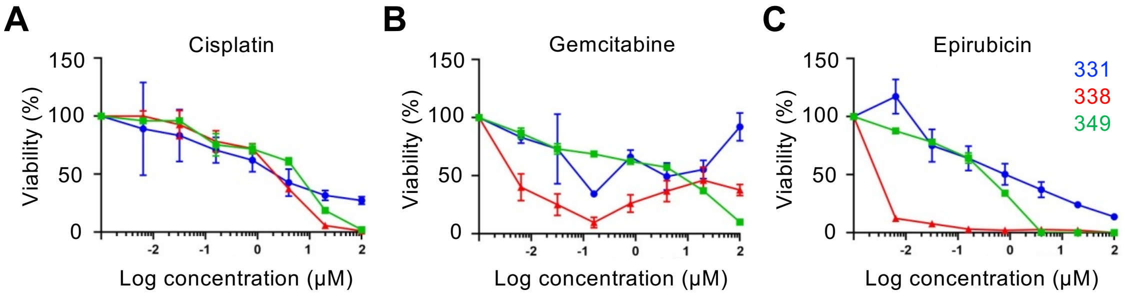

Supplementary Table S1. Clinicopathologic correlation of RRBP1 expression to clinical manifestations.

	RRBP1 high [a] (n = 142)	RRBP1 low (n = 55)	P value
Age (years)	66.9±11.7	66.0±8.2	0.557 [b]
Stage			
a/is	40 (28.1%)	19 (34.5%)	
1	39 (27.5%)	13 (23.6%)	0.266 [c]
2	25 (17.6%)	14 (25.5%)	
3	25 (17.6%)	8 (14.5%)	
4	13 (9.15%)	1 (1.8%)	
Multifocal tumor	37 (26.1%)	12 (21.8%)	0.537 [b]
High grade	132 (93.0%)	50 (90.9%)	0.627 [b]
Lymphovascular invasion	24 (16.9%)	9 (16.4%)	0.928 [b]
Carcinoma in situ	62 (43.7%)	21 (38.2%)	0.485 [b]
Papillary	124 (87.3%)	44 (80%)	0.193 [b]
Squamous differentiation	51 (35.9%)	14 (25.5%)	0.161 [b]
Distant metastasis	22 (15.5%)	3 (5.5%)	**0.046** [b]

[a] The IHC results of RRBP1 were derived from 197 UTUC patients whose tissues were arrayed in triplicate. The IHC intensity of UTUC <100 unit regarded as downregulation.

[b] By Chi-square test

[c] Kruskal Wallis test

Supplementary Table S2. Sequence of MS-PCR primer sets of RRBP1.

CpG site	Primer name	Sequence (5′ to 3′)	Length (bp)	Product size (bp)	Tm (℃)
Site 1	MPrimer_Site1_F	TTGTAAAGGAAAAATATTTTTTCGA	25	230	58
	MPrimer_Site1_R	GTAACACAATAAACAACCACGCA	23		60
	UPrimer_Site1_F	TTGTAAAGGAAAAATATTTTTTTGA	25	230	56
	UPrimer_Site1_R	ACCATAACACAATAAACAACCACAC	25		59
Site 2	MPrimer_Site2_F	TTTATTAAAGTGAGATATTGGTTTGC	26	149	54
	MPrimer_Site2_R	CTCCCTAAAAACTTACTCCTACGAC	25		72
	UPrimer_Site2_F	ATTAAAGTGAGATATTGGTTTGTGT	25	149	52
	UPrimer_Site2_R	CTCCCTAAAAACTTACTCCTACAAC	25		72
Site 3	MPrimer_Site3_F	GTAGGAGATATTGGAGTAGATTCGT	25	202	64
	MPrimer_Site3_R	TAAAAAAAATAAAAAAAACCGTCGA	25		68
	UPrimer_Site3_F	GTAGGAGATATTGGAGTAGATTTGT	25	202	64
	UPrimer_Site3_R	AAAAAAAATAAAAAAAACCATCAAC	25		72
